# The Destruction of the Anaerobic Environment Caused by Rumen Fistula Surgery Leads to Differences in the Rumen Microbial Diversity and Function of Sheep

**DOI:** 10.3389/fvets.2021.754195

**Published:** 2021-12-01

**Authors:** Yurong Cao, Baozhen Zhu, Fei Li, Duihong Zhang, Tongqing Guo, Fadi Li, Guo Yang

**Affiliations:** ^1^State Key Laboratory of Grassland Agro-Ecosystems, Key Laboratory of Grassland Livestock Industry Innovation, Ministry of Agriculture and Rural Affairs, Engineering Research Center of Grassland Industry, Ministry of Education, College of Pastoral Agriculture Science and Technology, Lanzhou University, Lanzhou, China; ^2^Northwest Institute of Eco-Environment and Resources, Chinese Academy of Sciences, Lanzhou, China

**Keywords:** rumen fistula surgery, microbial diversity, sheep, rumen microbial function, BugBase function prediction

## Abstract

This experiment was to study the impact of rumen fistula surgery on the rumen microbios in sheep. Six male adult *Hu* sheep (48.8 ± 0.23 kg, 0.5 years) were fed at 0700 and 1,800 with *ad libitum* access to water. The rumen fistula was installed in the same batch from 0600 to 0900. Monitoring the dry mater intake and the output of dry mater faces 1, 2, 4, 6, 8, 10, 12, 14 days after fistulated surgery. The collection of rumen fluid was arranged at 1d during rumen surgery (DRS1), 3d after rumen surgery (ARS3), and 14d after rumen surgery (ARS14) for volatile fatty acid (VFA) and DNA extraction for sequencing and bioinformatics analysis. There was no difference in DMI, the pH apparent digestibility of dry matter, organic matter, neutral detergent fiber, acid detergent fiber both before and 14 days after surgery. Increases were observed in the acetate and total VFA at ARS3. There was no difference in digestion of dry material, organic material, neutral detergent fiber, and acid detergent fiber before and after surgery. The relative abundance of *Bacteroides* decreased from 61.96% at DRS1 to 28.85% at ARS3. In comparison with the DRS1 and ARS3, the relative abundance of *Firmicutes* in the ARS14 increased to 44.58% (*P* < 0.01). *Proteobacteria* increased from 11.33% at DRS1 to 51.66% at ARS3 and then decreased to 11.39% at ARS14. *Prevotella* decreased form 61.06% at DRS1 to 28.04% in the ARS3. *Succinivibrio* increased from 8.32% at DRS1 to 48.58% at ARS3, but decreased to 10.43% in the ARS14. Compared with DRS1 and ARS3, the ARS14 was higher in the Simpson and Shannon index. As for the BugBase function prediction, rumen fistula surgery increased the microorganism abundance of aerobic and facultative anaerobic phenotype, and anaerobic phenotype was decreased in the ARS3. There was higher microorganism abundance of aerobic phenotype in the ARS14 than before fistula installation. In conclusion, the rumen fistula surgery destroys the anaerobic environment of rumen, leading to differences in rumen microbial diversity and function, but the apparent digestibility and total VFA were not affected.

## Introduction

Previous studies proposed that the feasibility of rumen fistula in animal experiments is based on the assumption that rumen fistula surgery does not impact the rumen function (1). Rumen fistula installation is a challenge for ruminants and requires a slit through the abdominal cavity and rumen, causing serious trauma (2, 3). As we all know, the rumen microbial community and protozoa undertake the digestion and metabolism of fiber, starch, protein, and other substances in an anaerobic environment (4). The destruction of the anaerobic environment is a disaster for the microorganisms exposed to the oxygen stress in the rumen after fistula surgery (5). Compared with the intact rumen, the rumen microbial diversity of the fistulated is reduced (6–8), and the total volatile fat acid (TVFA) increased and the pH decreased (9). The fistulated abdominal cavity owned a lower proportion of CH_4_, H_2_, and CO_2_, and a higher proportion of O_2_ and N_2_ in the rumen headspace gasses, which results in a decrease in energy utilization (8, 10).

On the other hand, the presence or absence of fistula surgery directly determines the sampling methods. Svga et al. (11) considered that the oral tube sampling is not a feasible alternative to fistula sampling to research the gas production and composition when comparing oral tube sampling to fistula sampling. In addition, there is little difference in profile of VFA (12) and microbial community because of the influence of saliva (13–15). However, some scientists concluded that there was no difference between oral tube sampling and fistula sampling, and it can adequately represent rumen fermentation parameters such as the pH, VFA profile, and mineral concentrations (16, 17).

The contribution of fistula animals cannot be denied, and they had been applied in ruminants for many decades (17). However, there is still controversy surrounding the question of whether the sample of rumen fistula animals really representatively reflects the purpose of the experiment for animal experiments. In practice, many animal trials and sampling methods leave these changes of microbial community wholly out of account. In some cases, comparing the sample of the oral tube and fistula on fistulated animals was equivalent to comparing with the sample of the fistulated or intact (15, 17, 18), which lacks sufficient evidence to provide proof. At present, figuring out the impact of fistula needs to unify three key variations: whether fistulated or not; whether it is the same group of animal before and after the fistula; whether a unified sampling method has been selected (oral tube or fistula). In addition, the different fistula methods (the one-step and two-step surgery) (19), individual differences, and species genetic backgrounds (15), may also be considered, but the impact of fistulation on destruction of rumen environment may be consistent.

Therefore, it is necessary to study the impact of rumen fistulation on the microbial community and the digestive physiology of ruminants. In this experiment, six sheep from the same batch were used to monitor the change before and after the fistulation, which could systematically reveal the effect of rumen fistulation on the microbial community and the digestive physiology of ruminants.

## Materials and Methods

### The Animal Feeding and Management

Six healthy adult male *Hu* sheep (mean weight: 48.8 ± 0.23 kg, 0.5 years), were selected from the Research Institute of Chinese Academy of Sciences, Lanzhou, China. The sheep were fed dry matter which was restricted to 3.5% of their body weight (7). The sheep were housed in metabolic cage and has *ad libitum* access to water. All sheep were fed the pelleted TMR [45% roughage and 55% concentrate supplements (Supplementary Table S1)] at 0800 and at 1,800.

### Surgery and Post-surgical

The surgical details of the procedure were based on the method reported by R. J. Komarek and Santra on sheep fistula (2, 20), the method of One-step installation was adopted by using T-shaped fistula (21). In addition, the post-operative care procedure referred to sheep fistula surgery concepts and techniques from Durmic's (22) study, of this second stage are described elsewhere. Some simple procedures are shown as follows. It is necessary to fully disinfect the surgical site and all instruments. The length of incision in the skin to the underlying layers was made large enough (4 cm) for the fistula. With the help of a wound retractor, the rumen was exposed, so that the rumen was visible. Rumen was pulled out from the incision by the gastric forceps gently. The assisting veterinarian fixed the rumen by two gastric forceps and two leaf forceps to avoid to slipping into the abdominal cavity. The rumen incision should be away from the main blood vessels on the rumen wall, then fixing the position with two gastric forceps. After the rumen incision was made. The wound retractor needed to be relieved. The silicone fistula was softened with hot water in advance, and then inserted into the rumen through the skin and rumen incision. After the fistula was reset, the rumen and skin were inlaid in the fistula. Finally, the purse string suture was sewed around the edge of the skin and rumen incision and drawn tight and tied around the neck of the fistula.

### Sample Collection and Test

Total fecal output and dry matter intake (DMI) was collected from each individual sheep every 2 days during 14 consecutive days for determining the change of the apparent digestibility of DMI (23). Restricted feeding was carried out on the 1st and 2nd days, and the digestibility results were invalid. The fecal samples were collected on the 1st day before the surgery and 14th day after the surgery. The BRS1 and ARS14 were collected separately to determine the apparent digestibility of organic matter (OM), neutral detergent fiber (NDF), and acid detergent fiber (ADF). The ash, OM, and DM was determined by complete combustion in automatic ash and drying analyzer (prepASH 340, Swiss). The measurement method of the NDF and ADF referenced Van Soest et al. (24) by fiber analyzer (ANKOM A200i fiber analyzer, USA). The sheep were weighed before and 14 days after the surgery. The rumen fluid was collected from [1 day during the rumen surgery (DRS1), 3 days after rumen surgery (ARS3) and 14 days after rumen surgery (ARS14)]. When sampling rumen fluid, the location and depth of the sample were identical. Sampling time was before feeding in the morning. The sample was filtered by four layers of cheesecloth and pH was immediately measured by pH-meter (Sartorius PB-10, Göttingen, Germany), and stored at −80°C until the DNA and VFA extraction. The concentrations of VFA in rumen fluid were measured by Gas Chromatography (GC) on a Thermo Fisher Trace 1300 GC system (Thermo Fisher Scientific, Milano, Italy) and the detailed method was described by Li et al. (25).

### DNA Extraction and 16S rRNA Sequencing and Bioinformatics Analysis

The DNA in rumen fluid samples was extracted by DNA extracted Kit (PowerSoil DNA Isolation Kit (Mobio Technologies Ins, Vancouver, Canada), and the detailed method described by Vandeventer et al. (26). According to the manufacturer's instructions, glass beads were added in the sample to mechanically lyse the microbial cells. After extraction, the concentration of DNA was determined by Spectrophotometry (NanoDrop™ 2000 Spectrophotometer, Massachusetts, USA), and the quality of DNA was evaluated by 2% gel electrophoresis. The electrophoresis results were quantified by utilizing software (Image J Software) and the sample was recovered and purified by 0.8X magnetic beads (Magic Pure Size Selection DNA Beads). After verifying the quality of DNA, the DNA was stored at −80°C, and was sent to 16S rRNA sequencing (Beijing Biomarker Technologies Co., Beijing, China) with dry ice. The amplicons were sequenced by utilizing single-molecule real-time sequencing (SMRT Cell) for 16S rRNA full-length to obtain the marker genes and the process was described by Schloss et al. (27). Making taxonomic annotations was based on Silva (Release132, http://www.arb-silva.de, SSU115) and abundance of microorganism reaching the 0.1% threshold will be distinguished and identified. Sequencing and sequence analysis are performed by qualified companies (Beijing Biomarker Technologies Co., Beijing, China).

### Statistical Analysis

The data of composition of rumen microbial, the pH, VFA and alpha diversity were analyzed by single factor repeated measures analysis (IBM SPSS statistics 20.0). All data are expressed as the mean and standard error. Significance was defined as *P* < 0.05 and < 0.01. Due to the uneven variance of rumen microbial data, data of rumen microbial composition needs to be log_10_ converted before single factor repeated measures analysis (17). The images of the BugBase feature prediction, Principal coordinate analysis plot (PcoA) provided by the platform of Biomarker Technologies Co after bioinformatics analysis (Beijing Biomec Biotechnology Co., China).

## Result

### Feed Intake and Nutrients Digestion

**Figure 1:**
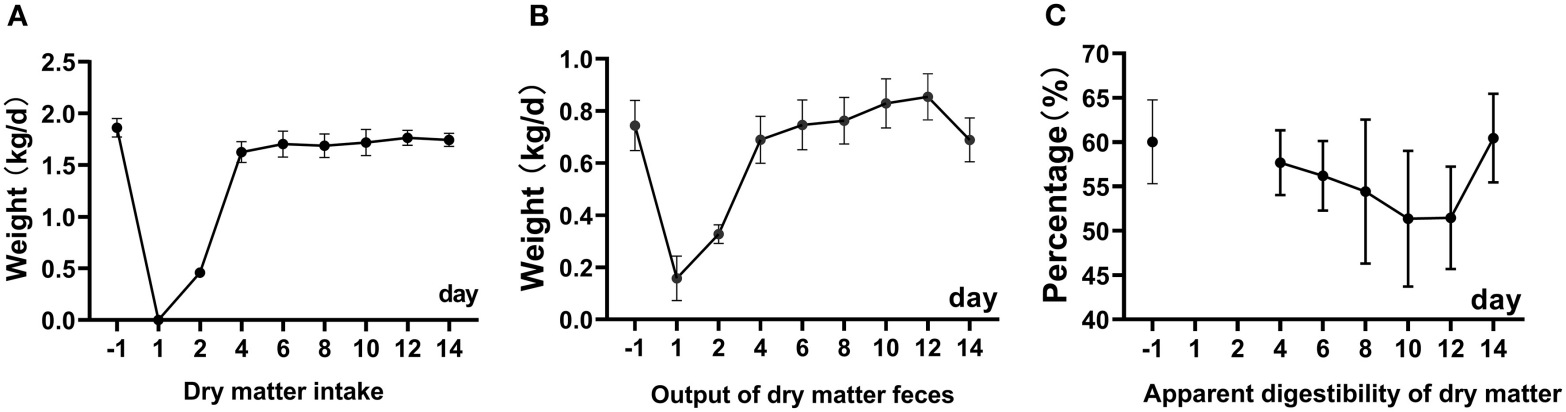
The picture shows the change of dry matter intake **(A)**, output of dry matter feces **(B)**, apparent digestibility of dry matter **(C)** within 14d after rumen fistula surgery. Restricted feeding was carried out on the 1th and 2th days, and the digestibility results were invalid **(C)**.

Before the surgery, the average DMI was 1.85 kg/d (Figure 1). The DMI was 0.5 kg/d on the second day and 1.8 kg/d on the 4th day after fistula surgery. Sheep were restricted to 0.5 kg on the 2nd day after the surgery to prevent excessive pressure in the rumen from affecting the stability of the fistula. On the third day, free feeding will be restored. From the 4th day to the 14th day, the DMI was within 1.65–1.8 kg/d. The output of feces dry mater gradually recovered to 0.6 kg/d from 1st to 4th days after the surgery and increased from 0.15 to 0.88 kg within 4 to 12 days after the surgery. The apparent digestibility of DM gradually decreased from 60.50 to 49.24% (the lowest point) within 12 days after the surgery, and increase to 58.50% on 14th days (Figure 1). There was no difference in the apparent digestibility of DM, OM, NDF, and ADF compared with BRS1 and ARS14 (Table 1).

**Table 1:** Changes in apparent digestibility before and after surgery.

**Items**	**BRS1**	**ARS14**	**SEM**	***P*-value**
DM, %	60.50	58.50	3.325	0.559
OM, %	66.96	65.16	3.670	0.569
NDF, %	38.06	36.93	3.186	0.763
ADF, %	32.15	29.75	3.578	0.521
BW, kg	48.80	51.18	2.043	0.278

*DM, dry mater; OM, organic matter; NDF, neutral detergent fiber; ADF, acid detergent fiber; BW, body weight*.

### Rumen Fermentation

In this experiment, compared with the DRS1, the pH value decreased by 0.27 in the ARS3 (*P* < 0.01, Table 2), and the DRS1 did not differ with the ARS14 (*P* > 0.05). Compared with the DRS1, the concentration of acetate and TVFA increased in the ARS3 (*P* < 0.01 and = 0.01), but the butyrate decreased in the ARS3 and ARS14 (*P* < 0.01). Compared with the ARS3, the concentration of acetate, TVFA decreased in the ARS14 (*P* < 0.05). Furthermore, the fistula surgery had no effect on the concentration of propionate, iso-butyrate, and A:P (acetate: propionate) among the sample times (*P* > 0.05). The concentration of valerate had increased tendency in the ARS3, and similar to the pre-operation level in the ARS14.

**Table 2:** The fermentation parameters of rumen fluid after fistula surgery in sheep.

**Items**	**DRS1^1^**	**ARS3^2^**	**ARS14^3^**	**SEM**	***P*-value**
pH	6.69^a^	6.45^b^	6.62^a^	0.036	<0.01
VFA (mmol/L)					
Acetate (A)	39.63^b^	53.39^a^	41.23^b^	2.130	<0.01
Propionate (P)	18.08	19.12	19.47	0.713	0.73
Isobutyrate	0.39	0.29	0.17	0.051	0.23
Butyrate	16.23^a^	11.33^b^	8.22^b^	1.061	<0.01
Isovalerate	0.60	0.86	0.28	0.118	0.13
Valerate	0.93	1.48	0.88	0.121	0.07
TVFA	75.85^b^	86.47^a^	70.26^b^	2.304	<0.01
A:P	2.25	2.85	2.20	0.154	0.15

1 *DRS1, 1d before rumen surgery;*

2 *ARS3, 3d after rumen surgery;*

3 *ARS14, 14d after rumen surgery*.

a, b *Values with different superscripts within a row differ significantly at P < 0.05*.

*TVFA, Total Volatile fatty acid; A:P, Acetate: Propionate*.

### Rumen Bacterial Diversity

**Figure 2:**
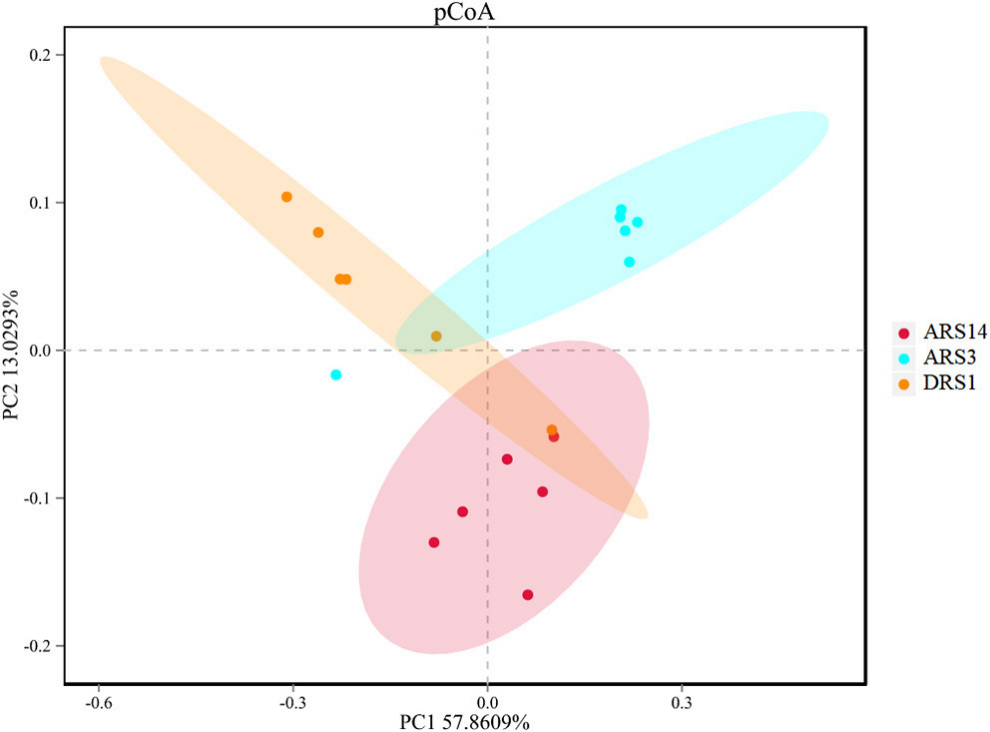
Principal coordinate analysis plot of total microbiota in rumen samples of 3 stages after rumen fistula surgery in sheep. PC1, principal component 1; PC2, principal component 2. DRS1, 1d during the rumen surgery; ARS3, 3d after the rumen surgery; ARS14, 14d after the rumen surgery.

Compared with the DRS1 and ARS3, the value of OTUs, Simpson and Shannon indexes (Table 3) in the ARS14 were higher (*P* < 0.02 and < 0.01 and = 0.01). The sequence number in the ARS3 was higher than those of the DRS1 and ARS14. The coverage of microbes in this investigation was ≥0.99 which indicated that the sequencing depth covers the sample size for most of the microbes. The PCoA analysis was made under the weighted-unifarc conditions in the OTUs level (Figure 2). The results showed that the first component approximately separated the DRS1, ARS3, and ARS14, which demonstrated the difference in rumen microbial diversity. The first principal component explained 57.37% of the variation and the second principal component explained 13.12% of the variation.

**Table 3:** The results of α diversity of microbes in rumen fluid after fistula surgery in sheep.

**Items**	**DRS1^1^**	**ARS3^2^**	**ARS14^3^**	**SEM**	***P*-value**
OTUs	90.0^b^	80.7^b^	138.2^a^	8.15	0.02
Seqs_Num	3,644	4,267	3,875	287.3	0.70
Simpson	3.19^b^	2.84^b^	4.70^a^	0.253	<0.01
Shannon	0.74^b^	0.70^b^	0.91^a^	0.032	0.01
Coverage	0.99	0.99	0.99	0.001	0.72

1 *DRS1, 1d before rumen surgery;*

2 *ARS3,3d after rumen surgery;*

3 *ARS14, 14d after rumen surgery*.

a, b *Values with different superscripts within a row differ significantly at P < 0.05*.

*OTUs, operational taxonomic units after Classification*.

### Rumen Microbial Community

The microbes at phylum-level (Table 4) that were >0.1% include 8 main phyla. The relative abundances of *Bacteroides* and *Firmicutes* accounted for more than 80% of the total rumen microbes in the DRS1 and ARS14. The relative abundance of *Bacteroides* decreased from 61.96% in the DRS1 to 28.85% in the ARS3 (*P* = 0.01), but cannot differ between in the ARS14 and ARS3 (*P* > 0.05). The relative abundance of *Firmicutes* in DRS1 was similar to the ARS3 (*P* > 0.05), but the ARS14 was higher than those in the DRS1 and ARS3 (*P* < 0.01). The relative abundance of *Proteobacteria* in in the ARS3 was higher than those in the DRS1 and ARS14 (*P* < 0.01), but cannot differ between in the ARS14 and the DRS1. In addition, the fistula surgery had no effect on the relative abundance of other phylum (*P* > 0.05).

**Table 4:** The composition of phylum-level microbes in rumen fluid after fistula surgery in sheep and their relative abundance.

**Phylum (%)**	**DRS1^1^**	**ARS3^2^**	**ARS14^3^**	**SEM**	***P*-value**
*Bacteroidota*	61.96^a^	28.85^b^	36.25^b^	4.983	0.01
*Firmicutes*	23.54^b^	17.00^b^	44.58^a^	3.643	<0.01
*Proteobacteria*	11.33^b^	51.66^a^	11.69^b^	6.097	<0.01
*Planctomycetota*	0.30	1.05	1.84	0.397	0.30
*Verrucomicrobiota*	0.00	0.05	1.57	0.473	0.32
*Spirochaetota*	0.23	0.03	1.16	0.256	0.16
*Actinobacteriota*	0.48	0.12	0.14	0.087	0.17
*Desulfobacterota*	0.10	0.06	0.12	0.019	0.46
*Others*	0.08	0.09	0.20	0.029	0.16
*Unclassified*	1.95	1.08	2.44	0.356	0.30

1 *DRS1, 1d before rumen surgery;*

2 *ARS3, 3d after rumen surgery;*

3 *ARS14, 14d after rumen surgery*.

a, b *Values with different superscripts within a row differ significantly at P < 0.05*.

The composition and relative abundance of micro-organisms that were >0.1% at the genus level in abundance (Table 5) are accounted for. The relative abundance of *Prevotella* in the DRS1 was lower than those in the ARS14 with the ARS3 (*P* < 0.01), but there was no difference between in the ARS14 and the ARS3 (*P* > 0.05). The relative abundance of *Succinivibrio* in the ARS3 was higher than those of the DRS1 and ARS14 (*P* < 0.01), but cannot differ in both the ARS14 and DRS1. In addition, the fistula surgery had no effect on the relative abundance of other genera (*P* > 0.05).

**Table 5:** The composition of genus-level microbes in rumen fluid after fistula surgery in sheep and their relative abundance.

**Genus (%)**	**DRS1^1^**	**ARS3^2^**	**ARS14^3^**	**SEM**	***P*-value**
*Prevotella*	61.06^a^	28.04^b^	32.17^b^	4.978	<0.01
*Succinivibrio*	8.32^b^	48.58^a^	10.43^b^	5.828	<0.01
*Ruminococcus*	10.00	7.42	9.78	2.426	0.65
*Succiniclasticum*	4.02	4.58	8.24	2.162	0.74
*Selenomonas*	3.28	2.36	4.42	0.954	0.13
*Ruminobacter*	0.27	1.77	0.01	0.898	0.33
*Gabonia*	0.46	0.46	1.40	0.239	0.16
*Anaerovibrio*	0.53	0.09	0.83	0.141	0.14
*Schwartzia*	0.67	1.42	0.41	0.328	0.31
*Quinella*	0.01	0.01	1.21	0.273	0.14
*Bact*	0.25	0.15	0.78	0.143	0.12
*Treponema*	0.23	0.03	1.16	0.256	0.19
*Clostridium*	0.05	0.03	0.57	0.122	0.14
*Succinimonas*	0.00	0.00	1.07	0.326	0.36
*Sharpea*	0.53	0.01	0.24	0.178	0.56
*Anaerocella*	0.00	0.02	0.52	0.146	0.30
*Anaerobiospirillum*	0.33	0.29	0.00	0.135	0.56
*Olsenella*	0.48	0.06	0.09	0.085	0.09
*Oscillibacter*	0.00	0.00	0.49	0.114	0.16
*Solobacterium*	0.08	0.06	0.24	0.048	0.20
*Sinibacillus*	0.00	0.00	0.57	0.192	0.43
*Desulfovibrio*	0.10	0.05	0.09	0.018	0.37
*Others*	0.38	0.57	1.02	0.227	0.54
*Unclassified*	8.95^b^	6.68^b^	24.28^a^	2.262	<0.01

1 *DRS1, 1d before rumen surgery;*

2 *ARS3, 3d after rumen surgery;*

3 *ARS14, 14d after rumen surgery*.

a, b *Values with different superscripts within a row differ significantly at P < 0.05*.

### BugBase Feature Prediction

**Figure 3:**
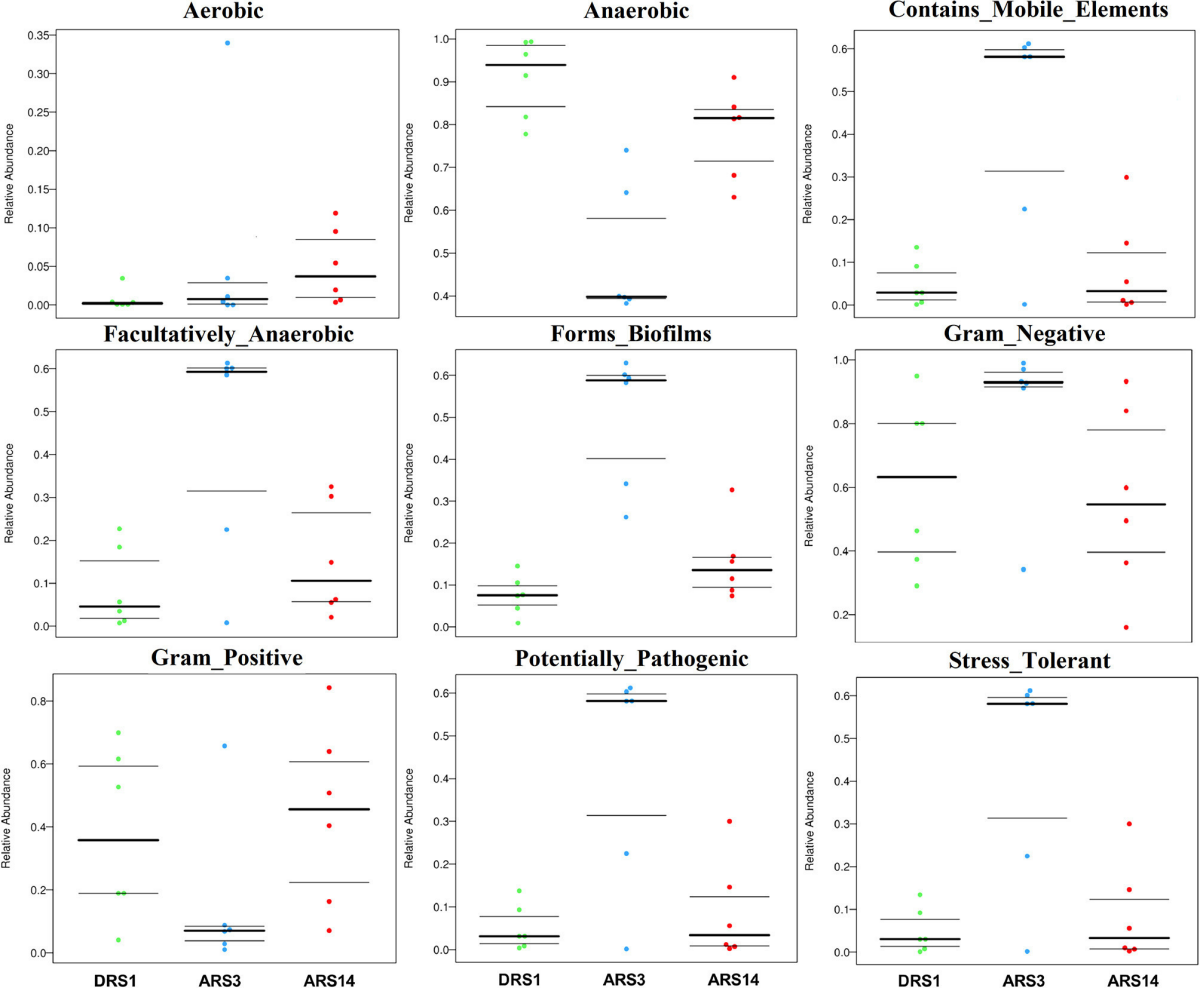
BugBase feature prediction include aerobic, anaerobic facultative anaerobic, contains mobile element content, potential pathogenicity, stress tolerance, forms biofirms and gram-negative feature. *DRS1*, 1d during the rumen surgery; *ARS3*, 3d after the rumen surgery; *ARS14*, 14d after the rumen surgery; f_: family, taxonomic unit. The datas are accessible in https://www.ncbi.nlm.nih.gov/, SUB9200751.

The results (Figure 3) show that, within 14 days after surgery, the microorganism abundance of aerobic phenotype was gradually increased among the stages after surgery. The microorganism abundance of anaerobic phenotype decreased in the ARS3 initially, followed by an increase in the ARS14; the microorganism abundance of the facultative-anaerobic, contains-mobile element, potential-pathogenicity, stress-tolerance, forms-biofirms phenotype was increased in the ARS3, and then decreased to close to the value of the DRS1 in the ARS14; the microorganism abundance of Gram-negative phenotype was increased in the ARS3 initially and then decreased in the ARS14, while the microorganism abundance of Gram-positive phenotype decreased in the ARS3 and then started to increase in the ARS14.

## Discussion

In this experiment, the DMI of sheep was not affected by fistula surgery, but the output of dry matter feces increased gradually, which resulted in a gradual decrease in DM digestibility until the 12th day (Figure 1). The output of dry matter feces decreased 14 days after fistula surgery. There was no difference in DM, OM, NDF, and ADF between before and 14 days after surgery (Table 1). Nevertheless, in terms of rumen microbes, we observed that the diversity of rumen microbes was increased and the relative abundance of *Prevotella* was decreased. The contradictory result may be attributed to two factors. The fistula surgery destroyed the pressure environment of the rumen and slowed down the peristalsis, to which led an increase in the retention time of the diet in the rumen (28), and it was conducive to rumen digestion. Likewise, the change in the composition of rumen microorganisms, the decrease in the relative abundance of *Prevotella*, and the increase in the *unclassified* microorganisms were observed, which influence the digestion activity of rumen microorganisms. From the results of previous studies, MacRae et al. (29) and Moate et al. (10) also observed no difference in the digestibility of DMI and DM on sheep and Holstein dairy cow in the ARS14. Furthermore, Hirayama et al. (28) observed that the fistula surgery reduced rumen pressure and rumen content passage rate on goats, thereby increasing the cumulative number of recovered particles in feces. From a numerical view in this experiment, the apparent digestibility of DM, OM, NDF, and ADF in the ARS14 were lower than those of the DRS1, but there was no difference. The phenomenon indicated that the influence of rumen microorganisms may be slightly greater than the slowing down of rumen peristalsis, which contributed to the conclusion that rumen fluid of rumen fistula animals cannot completely reflect the rumen fermentation function (11, 30). In summary, the rumen fistula surgery has no effect on the apparent digestibility of DM, OM, NDF, and ADF, and its rationalities are acceptable by judging from present results and previous studies.

The fermentation parameters are important indicators for determining the impact of fistula surgery on the rumen microbial function of sheep. In this experiment (Table 2), compared with the ARS14 and DRS1, the value of rumen pH and the concentration of butyrate were decreased (*P* < 0.05), the concentration of TVFA and acetate were increased (*P* < 0.05), as observed in the ARS3. However, there were no difference in rumen pH, the concentration of TVFA, and acetate between the ARS14 and DRS1. Consistent with our results, Wang et al. (7) also reported that no difference in rumen pH was observed at 14 days between the fistulated and the intact goat (*P* > 0.05). However, Rumsey et al. (6) reported that the rumen pH of the fistulated cattle was lower than that of the intact, and the TVFA was higher than that of the intact after 20 days. Wang et al. (8) compared the fermentation parameters of fistulated and intact cows, and the results were consistent with Rumsey et al. (6). The rumen peristalsis was decreased after fistula surgery, leading to an increase in the retention time of feed and a decrease in the pass rate of rumen (28). A study reported fully verified this results that considering the degree of rumen acidosis in fistula cow, the intact cows have a characteristic difference in oxygen utilization and nitrogen metabolism and suggested that the results of rumen fistula cows cannot reflect the normal metabolic process (30). Judging from VFA results in this experiment, it objectively reflects that the metabolism of rumen microbes is indeed affected by rumen fistula surgery.

Considering the result of rumen microbial diversity, the Shannon-wiener and Simpson indexes are higher in the ARS14 than those in the ARS3 and DRS1 (*P* < 0.05, Table 3) and the results of PcoA analysis suggested that there are differences in the diversity of rumen microbes in the three stages after surgery (Figure 2). Wang et al. (7) and Wang et al. (8) also reported that the ruminal microbial diversity of goats and cows received fistula surgery was lower than that of the intact goats and cows. The fistula surgery usually led considerable amounts of air to flow into the rumen headspace (10) and introduce the *unclassified* microbes into the rumen during the surgery, which could lead to the increase of microbial diversity. Moreover, in accordance with Wang et al. (8) who studied the gas compositions in the rumen by comparing the intact and fistulated cow and observed that the concentration of CO_2_, H_2_, and CH_4_ in fistula cattle are reduced and N_2_ and O_2_ are increased. Comprehensively, although the plug can prevent the unimpeded exchange of gas inside and outside of the rumen, it is inevitable that rumen fistula cause gas leakage. The destruction of the gas environment and the increase in the *unclassified* may be the reason for the change in diversity.

The different microbial diversity among the three stages may provide the evidence that the fistula surgery could change the rumen microbial composition in different taxonomic levels. In comparison with the DRS1, decrease in the relative abundance of *Bacteroidetes* was observed in the ARS3 and ARS14 (*P* < 0.05, Table 4). In comparison with the DRS1 and ARS3, the relative abundance of *Firmicutes* in the ARS14 increased to 44.58% (*P* < 0.05). The relative abundance of *Proteobacteria* in the ARS3 was higher than the ARS14 and DRS1 (*P* < 0.05). However, Wang et al. (7) reported that no differences in the relative abundance of *Firmicutes* and *Bacteroidetes* between the intact and fistulated goat after 2 weeks, but an increase in the relative abundance of *Proteobacteria* was observed (*P* < 0.001) (7). The similar relative abundance of *Firmicutes* and *Bacteroidetes* between the treatments mainly attributed to the different genotypes of rumen microorganisms carried by different species (8). Shin et al. (31) suggested that the increase of *Proteobacteria* is a distinctive signal for the occurrence of animal intestinal disorder and imbalance of dietary nutrition, which may indicate that a disorder of rumen microbes were caused by rumen fistula surgery.

Compared with the DRS1, the relative abundance of the *Prevotella* decreased (*P* < 0.05) nearly by 50% in the ARS3 and ARS14 (Table 5). However, Wang et al. (7) found no difference in the relative abundance of the *Prevotella* compared with the intact and the fistulated goat after 2 weeks. A study indicated that the increase by 5 fold in the level of recA mRNA was observed in response to conditions of oxidative stress when *Prevotella* was exposed to oxygen by removing the culture from the anaerobic box, and the recA genes are related to the stress response of cells when chromosomal DNA was severely damaged (32). The destruction of the anaerobic environment is the direct cause of the decrease of *Prevotella* in the present study. In addition, the DMI of sheep may be also the cause of the decrease in *Prevotella*. However, the relative abundance of *Prevotella* did not return to that before the operation in the ARS14, which may lead to the decrease in digestive activity of microorganisms in the ARS14.

Compared with the DRS1, the relative abundance of the *Succinivibrio* was increased in the ARS3 (*P* < 0.05) and then decreased in the ARS14 (*P* < 0.05, Table 5). The first reason was the decline in *Prevotella*, which caused the relative abundance of the *Succinivibrio* to increase. The second reason was the optimal carbon dioxide concentration and CO_2_ balance of the *Succinivibrio*. A study indicated that *Succinivibrio dextrinosolvens* grow well in 5% CO_2_ environment compared with 0% CO_2_, and the 5% CO_2_ flowing environment was contributed to the fixation of CO_2_ (33). The ratio of CO_2_ in the rumen headspace gas is 49.8%, and the dissolved CO_2_ was 69.7 mM before rumen fistula surgery, but the ratio of CO_2_ in the rumen headspace gasses was 13.4% after fistula surgery and the dissolved CO_2_ was 43.6 mM (8, 10). The high concentration of CO_2_ before the fistula surgery may inhibit the metabolic activity of the *Succinivibrio* (*Succinivibrio* produce CO_2_ as anaerobic metabolism). Moreover, the CO_2_ concentration after fistula surgery was closer to the optimum CO_2_ concentration of the *Succinivibrio* and the increases of air circulation rate promote the CO_2_ circulation. The third factor, and possibly the most likely factor, is that *Succinivibrio* has a wide selection of substrates, which can ferment dextrin, disaccharides and monosaccharides, and fructans (33), which makes it more energy-supplying than other microorganisms and better adapted to the rumen environment in the early stage of fistula surgery.

Furthermore, it should be noted that the relative abundance of un-classified microorganisms has increased. This result indicates that there was introduction of non-rumen microorganisms due to fistula surgery, but whether these non-rumen microorganisms are related to apparent digestibility, it cannot be determined due to the limitations of the sequencing data. According to microbial structure and apparent digestibility, this experiment could be read that the main factors affecting rumen digestibility are still concentrated in the obligated anaerobic (*Firmicutes*), such as the decrease of *Prevotella* (participating in synergistically digestion of fiber and starch) caused the continued impact on rumen digestive function in the ARS14. Considering the structure and diversity of microorganisms, a series of studies agreed that oral tube sampling and fistula sampling (fistula and non-fistula) can be substituted for each other in the composition and diversity of rumen microbes, and the representative samples can also be obtained (12, 17, 34). However, some scientists have made completely the opposite conclusions (11, 30), and suggested that the rumen fluid of fistula animals can not reflect the condition of intact animals. In summary, whether the fistula and intact sampling can replace each other may require reconsideration of the impact of rumen fistula surgery.

Through the analysis of the BugBase Feature Prediction, the changes in rumen microbial function have been further verified. In this trial, the microorganism abundance of aerobic and facultative anaerobic increased, and the microorganism abundance of anaerobic phenotypes decreased in the ARS3 (Figure 3). The study showed that the fistula surgery destroys the rumen anaerobic environment, resulting in the air enter the rumen along with the fistula moving, the activity of obligate anaerobic was impacted by the imbalance of rumen anaerobic environment (8, 10, 14). In addition, the changes in the microorganism abundance of contains-mobile element, form-biofilm and potential-pathogenic phenotypes increased in the ARS3 and then declined in the ARS14, which demonstrated that some rumen microbial community and function trend to become active after fistula surgery, then gradually forms a new balance as the internal environment becomes stable. The microorganism abundance of gram-negative and gram-positive phenotypes showed completely opposite results after fistula surgery. In addition, the microorganism abundance of stress-tolerant phenotype showed that rumen fistula surgery has caused stress in the ARS3. A study conducted by Dahl et al. (35) illustrated that the outer membrane-lipopolysaccharide portion of the gram-negative cell wall initially protects the bacteria from extracellular singlet oxygen, which further verified the existence of oxygen stress in the rumen of a fistulated animal. The microorganism abundance of stress-tolerant phenotype in the ARS14 was declined in those before the surgery, but the results of aerobic phenotype, anaerobic phenotype, and facultative anaerobic phenotype did not seem to be considered as recovery, or there was no difference. The acid-base balance of experimental acidosis given in the fistulated and intact illustrated that the intact cows have more pronounced and longer duration of acidosis caused by the administration of sucrose to the rumen and the trial of the fistulated cows do not reflect the normal metabolic process and characteristic differences were observed in oxygen utilization and nitrogen metabolism (30). All in all, the results of the BugBase feature prediction turn out that the function of rumen microorganism has experienced fluctuations due to the damage of rumen anaerobic environment, and then rebalance existed in this process, but whether the new balance can be equivalent to the balance of the intact needs to be further verified by experiments.

## Conclusion and Prospect

The present results illustrated that the rumen fistula surgery increased microbial diversity, and led to the decrease of *Prevotella* (*Bacteroidota*) 3 and 14 days after fistula surgery and the increase of *Succinivibrio* (*Proteobacteria*) 3 days after fistula surgery. In addition, the rumen fistula surgery leads to an increase of the microorganism abundance of aerobic, stress-tolerance, facultative anaerobic phenotype, and a decrease in anaerobic phenotype. However, there is no difference in DMI, apparent digestibility of DM, OM, NDF, ADF, pH, and TVFA both before and 14 days after surgery. The rumen fluid of fistula animals in microbiota research may not be representative of the intact animals, which requires further detailed functional analysis and identification.

## Data Availability Statement

The datasets presented in this study can be found in online repositories. The names of the repository/repositories and accession number(s) can be found below: https://www.ncbi.nlm.nih.gov/, SUB9200751.

## Ethics Statement

The animal study was reviewed and approved by the Institutional Animal Care and Use Committee of Lanzhou University. Written informed consent was obtained from the owners for the participation of their animals in this study.

## Author Contributions

YC and FeL: conceptualization, investigation, and writing—review and editing. BZ, DZ, and TG: validation. GY: resources. YC: data curation and writing—original draft preparation. FaL: supervision. FaL and FeL: project administration and funding acquisition. All authors have read and agreed to the published version of the manuscript.

## Funding

The research was financially supported by the National Natural Science Foundation of China, grant number: 32072754, and the Program for Changjiang Scholars and Innovative Research Team in University, grant number: IRT_17R50.

## Conflict of Interest

The authors declare that the research was conducted in the absence of any commercial or financial relationships that could be construed as a potential conflict of interest.

## Publisher's Note

All claims expressed in this article are solely those of the authors and do not necessarily represent those of their affiliated organizations, or those of the publisher, the editors and the reviewers. Any product that may be evaluated in this article, or claim that may be made by its manufacturer, is not guaranteed or endorsed by the publisher.

## References

[1] Hayes BW Little CO Mitchell GE. Influence of ruminal, abomasal and intestinal fistulation on digestion in steers. J Anim Sci. (1964) 23:764. 10.2527/jas1964.233764x

[2] Santra A Karim SA. Rumen cannulation in sheep and goats: fabrication of cannula and surgical procedure for its implantation. Indian J Anim Sci. (2002) 72:978–80.

[3] Prasad V Devarathnam J Mahesh R Sumiran N. Surgical management of traumatic ruminal fistula in an Ongole cow. Int J Livest Res. (2014) 4:43–6. 10.5455/ijlr.20140711114927

[4] Khafipour E Li S Plaizier JC Krause DO. Rumen microbiome composition determined using two nutritional models of subacute ruminal acidosis. App Environ Microbiol. (2009) 75:7115–24. 10.1128/AEM.00739-09 19783747 PMC2786511

[5] Shen JS Chai Z Song LJ Liu JX Wu YM. Insertion depth of oral stomach tubes may affect the fermentation parameters of ruminal fluid collected in dairy cows1. J Dairy Sci. (2012) 95:5978–84. 10.3168/jds.2012-5499 22921624

[6] Rumsey TS Putnam PA Williams EE Samuelson G. Effect of ruminal and esophageal fistulation on ruminal parameters, saliva flow, ekg patterns and respiratory rate of beef steers. J Anim Sci. (1972) 35:1248–56. 10.2527/jas1972.3561248x 4647455

[7] Wang L Wu D Yan T Wang L. The impact of rumen cannulation on the microbial community of goat rumens as measured using 16S rRNA high-throughput sequencing. J Anim Physiol Anim Nutr. (2018) 102:175–83. 10.1111/jpn.12676 29057500

[8] Wang R Wang M Zhang X Wen J Ma Z Long D. Effects of rumen cannulation on dissolved gases and methanogen community in dairy cows. J Dairy Sci. (2019) 102:2275–82. 10.3168/jds.2018-15187 30692015

[9] Kim E Kim SB Baek YC Min SK Choe C Yoo JG. Effects of rumen cannulation surgery on physiological parameters and rumen fluid pH in Korean native Hanwoo cattle. Korean J Vet Serv. (2018) 41:221–8. 10.7853/kjvs.2018.41.4.221

[10] Moate PJ Williams SRO Deighton MH Jacobs JL Wales WJ of Conference. Influence of rumen cannulation on feed intake, milk production, enteric methane production and composition of rumen headspace gas. In: Advances in Animal Bioscience, Greenhouse Gases & Animal Agriculture Conference (Dublin) (2013).

[11] Svga B Fs B Jwc B Jd B Wfp B. In dairy cattle, the stomach tube method is not a feasible alternative to the rumen cannulation method to examine *in vitro* gas and methane production. Anim Feed Sci Technol. (2019)256:4259.

[12] Terre M Castells L Fabregas F Bach A. Short communication: Comparison of pH, volatile fatty acids, and microbiome of rumen samples from preweaned calves obtained via cannula or stomach tube. J Dairy sci. (2013) 96:5290–4. 10.3168/jds.2012-5921 23706486

[13] Geishauser T Gitzel A. A comparison of rumen fluid sampled by oro-ruminal probe versus rumen fistula. Small Rumin Res. (1996) 21:63–9. 10.1016/0921-4488(95)00810-1

[14] Paz HA Anderson CL Muller MJ Kononoff PJ Fernando SC. Rumen bacterial community composition in holstein and jersey cows is different under same dietary condition and is not affected by sampling method. Front Microbiol. (2016) 7:1206. 10.3389/fmicb.2016.01206 27536291 PMC4971436

[15] Lage C Risnen SE Melgar A Nedelkov K Hristov AN. Comparison of two sampling techniques for evaluating ruminal fermentation and microbiota in the Planktonic Phase of Rumen Digesta in Dairy Cows. Front Microbiol. (2020) 11:618032. 10.3389/fmicb.2020.618032 33424820 PMC7785721

[16] Lodge-Ivey JB-S Horvath MB. Technical note: bacterial diversity and fermentation end products in rumen fluid samples collected via oral lavage or rumen cannula. J Anim Sci. (2009) 87:2333–7. 10.2527/jas.2008-1472 19329475

[17] Ramos-Morales E Arco-Pérez A Martín-García I Yáñez-Ruiz D Frutos P Hervás G. Use of stomach tubing as an alternative to rumen cannulation to study ruminal fermentation and microbiota in sheep and goats. Anim Feed Sci Tech. (2014) 198:57–66. 10.1016/j.anifeedsci.2014.09.016

[18] Castillo C Hernández J. Ruminal fistulation and cannulation: a necessary procedure for the advancement of biotechnological research in Ruminants. Animals. (2021) 7:1870–83. 10.3390/ani11071870 34201623 PMC8300264

[19] Gleason CB White RR Schramm HH. Influence of two rumen cannulation techniques on postoperative recovery in sheep. Vet. Surg. (2020) 50:312–22. 10.1111/vsu.13547 33336847

[20] Komarek RJ. Rumen and abomasal cannulation of sheep with specially designed cannulas and a cannula insertion instrument. J Anim Sci. (1981) 3:790–5. 10.2527/jas1981.533790x 7319954

[21] Girardi A Sousa S Sabes A Bueno G Módolo T Bonacin Y. One-stage rumen fistulation with permanent silicone cannula in sheep. Nucleus Animalium. (2017) 9:91–6. 10.3738/21751463.2140

[22] Durmic Z Mcgrath P Wilmot M Adams N Tan T Callahan L. Surgical and postoperative events during permanent fistulation of sheep rumen by the Schalk and Amadon method. Aust Vet J. (2015) 93:234–9. 10.1111/avj.12343 26113348

[23] Martin C Rouel J Jouany JP Doreau M Chilliard Y. Methane output and diet digestibility in response to feeding dairy cows crude linseed, extruded linseed, or linseed oil. J Anim Sci. (2008) 86:2642–50. 10.2527/jas.2007-0774 18469051

[24] Van Soest PJ Robertson JB Lewis BA. Methods for dietary fiber, 484 neutral detergent fiber, and nonstarch polysaccharides in relation to animal 485 nutrition. J Dairy Sci. (1991) 74:3583–97. 10.3168/jds.S0022-0302(91)78551-2 1660498

[25] Li F Wang Z Dong C Li FD Wang W Yuan Z. Rumen bacteria communities and performances of fattening lambs with a lower or greater subacute ruminal acidosis risk. Front Microbiol. (2017) 8:2506. 10.3389/fmicb.2017.02506 29312208 PMC5733016

[26] Vandeventer PE Weigel KM Salazar J Erwin B Niemz A. Mechanical disruption of lysis-resistant bacterial cells by use of a miniature, low-power, disposable device. J Clin Microbiol. (2011) 49:2533–9. 10.1128/JCM.02171-10 21543569 PMC3147885

[27] Schloss PD Jenior ML Koumpouras CC Westcott SL Highlander SK. Sequencing 16S rRNA gene fragments using the PacBio SMRT DNA sequencing system. PeerJ. (2016) 4:e1869. 10.7717/peerj.1869 27069806 PMC4824876

[28] Hirayama T Katoh K. Effects of fistula size on rumen internal pressure and passage rate of feed in goats. Small Rumin Res. (2005) 56:277–80. 10.1016/j.smallrumres.2004.06.004

[29] MacRae JC Wilson S. The effects of various forms of gastrointestinal cannulation on digestive measurements in sheep. Br J Nutr. (1977) 38:65–71. 10.1079/BJN19770062 889773

[30] HekBasz Z Nicpo DJ. Acid base equilibrium in experimental acidosis of cows with and without rumen fistula. Acta Physi Pol. (1977) 28:77–84. 17264

[31] Shin NR Whon TW Bae J-W. Proteobacteria: microbial signature of dysbiosis in gut microbiota. Trends Biotechnol. (2015) 33:496–503. 10.1016/j.tibtech.2015.06.011 26210164

[32] Aminov RI Tajima K Ogata K Nagamine T Sugiura M Benno Y. Transcriptional regulation of the prevotella ruminicola reca gene. Curr Microbiol. (1998) 36:259–65. 10.1007/s002849900306 9541560

[33] O'Herrin SM Kenealy WR. Glucose and carbon dioxide metabolism by *Succinivibrio dextrinosolvens*. Appl Environ Microbiol. (1993) 59:748–55. 10.1128/aem.59.3.748-755.1993 8481001 PMC202185

[34] Song J Choi H Jeong JY Lee S Kim M. Effects of sampling techniques and sites on rumen microbiome and fermentation parameters in hanwoo steers. J Microbiol Biotechn. (2018) 28:1700–5. 10.4014/jmb.1803.03002 29996593

[35] Dahl TA Midden WR Hartman PE. Comparison of killing of gram-negative and gram-positive bacteria by pure singlet oxygen. J Bacteriol. (1989) 171:2188–94. 10.1128/jb.171.4.2188-2194.1989 2703469 PMC209876

