# Supplementary material for: The Destruction of the Anaerobic Environment Caused by Rumen Fistula Surgery Leads to Differences in the Rumen Microbial Diversity and Function of Sheep
Source: Front Vet Sci. 2021 Dec 1;8:754195. doi: 10.3389/fvets.2021.754195 (PMC8671607; doi:10.3389/fvets.2021.754195)
Supplement: Supplementary file 1 [file Table_1.DOCX]

**SUPPLEMENTARY MATERIALS**

Table S1 the composition and ingredients of the pelleted TMR contain

| item | |  | | |
| --- | --- | --- | --- | --- |
| Ingredient | | g/kg of DM | |  |
| corn | | 300.0 | |  |
| corn straw | | 270.0 | |  |
| alfalfa | | 180.0 | |  |
| soybean meal | | 70.0 | |  |
| cotton seed meal | | 60.0 | |  |
| corn hull | | 50.0 | |  |
| molasses | | 40.0 | |  |
| limestone | | 12.0 | |  |
| sodium chloride | | 7.0 | |  |
| expanded urea | | 6.0 | |  |
| Premix^1^ | | 5.0 | |  |
| Chemical composition | | % | |  |
| CP | | 14.74 | |  |
| NDF | | 30.61 | |  |
| ADF | | 19.47 | |  |
| ASH | | 8.26 | |  |
| Ca | | 10.5 | |  |
| P | | 0.57 | |  |
| NE (MJ/kg of DM) ^2^ | | 6.98 | | |

1.The premix provided the following per kg of diets: VA 1500 IU; VD 160 IU; VE 18 IU; Co 0.30mg; Cu 18 mg; I 1.5mg; Fe 70 mg; Mn 38 mg; Se 0.28 mg; Zn 40 mg.

2.Calculated from the analyzed value of the dietary ingredients.
